# Trimethylamine-N-Oxide (TMAO) Predicts Cardiovascular Mortality in Peripheral Artery Disease

**DOI:** 10.1038/s41598-019-52082-z

**Published:** 2019-10-30

**Authors:** Carmen Roncal, Esther Martínez-Aguilar, Josune Orbe, Susana Ravassa, Alejandro Fernandez-Montero, Goren Saenz-Pipaon, Ana Ugarte, Ander Estella-Hermoso de Mendoza, Jose A. Rodriguez, Sebastián Fernández-Alonso, Leopoldo Fernández-Alonso, Julen Oyarzabal, Jose A. Paramo

**Affiliations:** 1 Laboratory of Atherothrombosis, Program of Cardiovascular Diseases, CIMA Universidad de Navarra, Pamplona, Spain; 2 CIBERCV, Instituto de Salud Carlos III, Madrid, Spain; 3 IdiSNA, Pamplona, Spain; 4 Departamento de Angiología y Cirugía Vascular, Complejo Hospitalario de Navarra, Pamplona, Spain; 5 Laboratory of Heart Failure, Program of Cardiovascular Diseases, CIMA Universidad de Navarra, Pamplona, Spain; 6 Department of Occupational Medicine, Clínica Universidad de Navarra, Pamplona, Spain; 7 Small Molecules Platform, Program of Molecular Therapies, CIMA Universidad de Navarra, Pamplona, Spain; 8 Hematology Service, Clínica Universidad de Navarra, Pamplona, Spain

**Keywords:** Risk factors, Outcomes research, Prognostic markers

## Abstract

Peripheral artery disease (PAD) is a major cause of acute and chronic illness, with extremely poor prognosis that remains underdiagnosed and undertreated. Trimethylamine-N-Oxide (TMAO), a gut derived metabolite, has been associated with atherosclerotic burden. We determined plasma levels of TMAO by mass spectrometry and evaluated their association with PAD severity and prognosis. 262 symptomatic PAD patients (mean age 70 years, 87% men) categorized in intermittent claudication (IC, n = 147) and critical limb ischemia (CLI, n = 115) were followed-up for a mean average of 4 years (min 1-max 102 months). TMAO levels were increased in CLI compared to IC (P < 0.001). Receiver operating characteristic (ROC) curves for severity (CLI) rendered a cutoff of 2.26 µmol/L for TMAO (62% sensitivity, 76% specificity). Patients with TMAO > 2.26 µmol/L exhibited higher risk of cardiovascular death (sub-hazard ratios ≥2, P < 0.05) that remained significant after adjustment for confounding factors. TMAO levels were associated to disease severity and CV-mortality in our cohort, suggesting an improvement of PAD prognosis with the measurement of TMAO. Overall, our results indicate that the intestinal bacterial function, together with the activity of key hepatic enzymes for TMA oxidation (FMO3) and renal function, should be considered when designing therapeutic strategies to control gut-derived metabolites in vascular patients.

## Introduction

Peripheral artery disease (PAD) is a largely unrecognized manifestation of atherosclerotic pathologies and a major cause of acute and chronic illness. Its prevalence in Western societies increases with age; 20% of the patients over 65 years are diagnosed with PAD, and is associated with exceptionally high risk for myocardial infarction (MI), ischemic stroke and death. Given that PAD typically presents in later life, its prevalence will undoubtedly escalate in response to changing population demographics and lifestyle^1,2^. Despite this prospect and its extremely poor prognosis, PAD remains undiagnosed and undertreated, highlighting the need for new markers that may provide insight into the underlying pathophysiology, improve long-term clinical risk prediction, and suggest novel therapeutic targets.

Recent studies have shown a direct link between dietary nutrients, intestinal microbiota, and cardiometabolic diseases^3,4^. In this regard, several human and animal studies have identified trimethylamine N-oxide (TMAO), metabolite of the gut microbe-derived trimethylamine (TMA), as a potential promoter of chronic diseases including atherosclerosis in humans^5–7^. TMA is generated by the action of specific microbial enzymes on dietary nutrients (choline, phosphatidylcholine, and L-carnitine), then absorbed by the host and converted into TMAO by hepatic flavin monooxygenases (FMO3). Subsequently, TMAO is either transported to the tissues for accumulation as an osmolyte or, more commonly, cleared by the kidney^8^. In humans circulating TMAO and its precursors have been associated with atherosclerotic burden^9,10^, increased risk for cardiovascular (CV) disease, and Major adverse CV events (MACE) and death in coronary and peripheral arterial diseases^5–7,11–14^. In preclinical models TMAO accelerated atherosclerosis development^5,11,15^, and induced thrombosis^12,16^, supporting a role for gut microbiota in the pathogenesis of atherosclerosis and its associated complications.

Taking in consideration the need of new markers for CV risk evaluation in PAD and the association of TMAO with vascular pathologies and worse outcome, we hypothesized that the measurement of TMAO could help to assess outcome evaluation in symptomatic PAD patients.

## Results

### TMAO levels are increased in CLI patients

Table 1 summarizes the demographic and clinical parameters of PAD patients (n = 262). First we analysed the association of TMAO with well-established vascular risk factors in PAD patients. A positive correlation with age (r = 0.25, p < 0.001) and hs-CRP (r = 0.22, p = 0.001) could be demonstrated, while an inverse association was found with ankle brachial index (ABI, r = −0.23, p = 0.001), estimated-glomerular filtration rate (eGFR, r = −0.40, p < 0.001), and HDL-C (r = −0.18, p = 0.003). Similarly, correlations between TMAO and age (r = 0.307, p = 0.040) and eGFR (r = −0.384, p = 0.009) were also observed in a smaller group of people with no manifest CV disease (n = 45, Supplementary Table 2). PAD patients were then categorized according to disease severity (Table 1). Subjects with critical limb ischemia (CLI) were older, presented higher percentage of diabetes and chronic kidney disease (CKD), and lower eGFR compared to intermittent claudication (IC). Levels of hepatic enzymes (AST, ALT and GGT) were similar and within usual ranges in both groups indicating normal liver function in our cohort. TMAO determination showed increased levels of TMAO in CLI subjects compared to IC [median: 1.30(0.83–2.25) IC vs 2.77(1.45–7.16) µmol/L CLI, p < 0.001].

**Table 1:** Demographic and clinical parameters in PAD patients (n = 262).

	PAD (n = 262)	IC n = 147	CLI n = 115	p *vs* IC
*Demographic and clinical data*				
Sex (male, %)	87	88	85	0.442
Age (years)	70 (11)	68 (10)	73 (11)	<0.001
Smokers (%)				
Never	20	12	29	0.004
Current	32	35	29	
Former	48	53	42	
Diabetes mellitus (%)	53	36	74	<0.001
Hypertension (%)	74	72	77	0.331
Dyslipidemia (%)	62	67	57	0.093
BMI (kg/m^2^)	28 (5)	28 (5)	28 (6)	0.830
ABI	0.55 (0.19)	0.62 (0.17)	0.38 (0.13)	<0.001
*Personal history (%)*				
COPD	14	14	13	0.772
CKD	39	24	58	<0.001
AMI	28	26	30	0.486
Cardiomyopathy	25	14	38	<0.001
Cerebral ischemia	9	5	14	0.009
*Treatment (%)*				
Anticoagulants	13	8	20	0.005
Antiplatelets	77	82	70	0.033
ACE inhibitors	34	32	36	0.440
ARA-2	27	23	32	0.102
Calcium antagonists	22	18	27	0.071
Vasodilators	7	6	8	0.589
β-Blockers	24	24	23	0.722
Statins	65	69	59	0.084
*Laboratory data*				
Total col (mg/mL)	172 (46)	187 (41)	153 (47)	<0.001
LDL-C (mg/dL)	110 (91)	114 (83)	104 (99)	0.360
HDL-C (mg/dL)	43 (16)	49 (15)	36 (13)	<0.001
Triglycerides (mg/dL)	145 (81)	151 (88)	138 (70)	0.194
hs-CRP^a^ (mg/mL)	5 (11)	3 (4)	10 (22)	<0.001
AST (U/L)	21 (9)	21 (9)	20 (8)	0.194
ALT (U/L)	21 (14)	22 (14)	19 (13)	0.081
GGT (U/L)	54 (69)	50 (61)	60 (78)	0.244
eGFR (mL/min/1.73 m^2^)	73 (21)	79 (19)	66 (22)	<0.001

Mean (SD) is shown. ^a^Log-transformed variables are presented as median (interquartile range). BMI: body mass index, ABI: ankle-brachial index, COPD: chronic obstructive pulmonary disease, CKD: chronic kidney disease, AMI: acute myocardial infarction, ACE: angiotensin-converting enzyme, ARA-2: angiotensin II receptor antagonist, LDL: low-density lipoprotein, HDL: high-density lipoprotein. ALT: alanine aminotransferase; AST: aspartate aminotransferase; GGT: gamma glutamyltransferase; GFR: Glomerular filtration rate.

Logistic regression analysis showed and association between TMAO and PAD severity alone (model 1, Table 2) and after correcting by other risk factors (model 2, Table 2). The interaction between eGFR and TMAO in PAD severity was excluded by logistic regression analysis (p for interaction 0.632).

**Table 2:** Logistic regression analysis to estimate the odds ratio (OR, 95% confidence interval) for TMAO in PAD patients (n = 262). Dependent variable IC/CLI.

	*TMAO, µmol/L* ^a^		*TMAO, cutoff (>2.26 µmol/L)*		
		p	≤2.26	>2.26	p
*Model 1*	1.84 (1.49–2.27)	<0.001	1	4.97 (2.92–8.47)	<0.001
*Model 2*	1.68 (1.3–2.2)	<0.001	1	4.4 (2.1–9.1)	<0.001

^a^Log-transformed variable. Unadjusted Model 1. Model 2: sex, age, smoking, diabetes mellitus, hypertension, dyslipidemia, HDL-C, eGFR (<60 mL/min/1.73 m^2^), and hs-CRP (log-transformed).

Receiver operating characteristic (ROC) curves were plotted to assess disease severity (IC vs. CLI) for TMAO [AUC: 0.731 ± 0.032 (95% CI: 0.669–0.792) p < 0.001] rendering a cut-off value of >2.26 µmol/L (62% sensitivity, 76% specificity), that was latter used to performed similar multivariate analysis. High TMAO levels (>2.26 µmol/L, n = 107) presented a significant association with PAD severity alone and after adjusting by other covariates (Table 2).

### TMAO is associated to CV-mortality in PAD

To evaluate the possible prognostic value of TMAO we recorded MACE (n = 135) and mortality, either all-cause (n = 101) or cardiovascular (n = 39) during the follow-up (4 years, min 1-max 102 months).

Cox regression analyses for overall mortality were performed before and after covariate adjustment. High TMAO concentrations were associated to all-cause mortality in the unadjusted model, but not after correcting by traditional risk factors (Supplementary Table 3).

**Figure 1:**
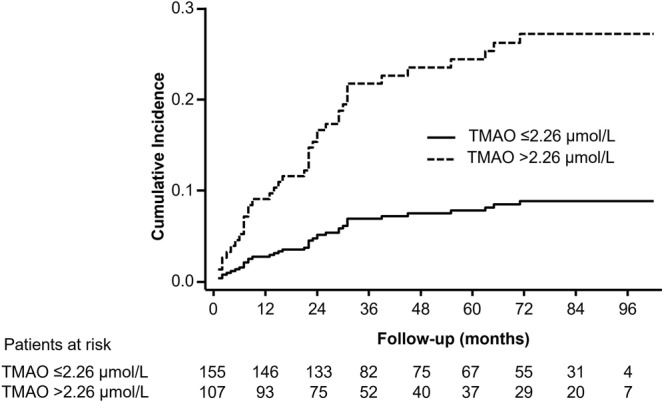
TMAO levels are associated to CV-mortality in PAD. Unadjusted cumulative incidence curve for the risk of CV mortality after a competing risk analysis (Fine-Gray model), where the competing event was non-CV death, in all patients categorized according to the TMAO cutoff (>2.26 µmol/L).

We next determined the prognostic value of TMAO for CV-mortality. High TMAO levels rendered significant associations with all tested models (Table 3). Correspondingly, categorized TMAO displayed similar association (>2.26 µmol/L, Fig. 1), with sub-hazard ratios above 2 for all tested models, being highest for model 4 (Table 3).

**Table 3:** Association of TMAO (µmol/L) with CV death.

	*TMAO, µmol/L* ^a^			*TMAO, cutoff (>2.26 µmol/L)*		
	SHR	95% CI	p	SHR	95% CI	p
CV death						
Model 1	1.52	1.27–1.83	<0.001	3.44	1.74–6.79	<0.001
Model 2	1.39	1.13–1.70	0.001	2.55	1.22–5.28	0.012
Model 3	1.29	1.05–1.60	0.015	2.15	1.04–4.48	0.040
Model 4	1.52	1.27–1.82	<0.001	3.36	1.68–6.70	0.001

^a^Log-transformed variable. Sub-hazard ratios (SHR) are effects sizes for a doubling of TMAO in plasma. Model 1: unadjusted. Model 2: sex, age and hs-CRP (log). Model 3: diabetes mellitus, hypertension, and eGFR (<60 mL/min/1.73 m^2^). Model 4: smoking, dyslipidemia, HDL-C.

Finally, we determined proportional hazards for TMAO considering MACE (n = 135, 51%). TMAO showed no independent association with this outcome in the multivariate Cox analysis (Supplementary Table 3).

To estimate the potential of TMAO to improve CV-death risk prediction over and beyond the basal models considered, Harrell’s C, integrated discrimination improvement (IDI) and continuous NRI analyses were performed. The addition of the categorical variable TMAO (>2.26 µmol/L) to the considered models including the previously mentioned relevant covariates, improved risk prediction for CV mortality in symptomatic PAD patients as assessed by NRI in models 2, 3 and 4 (Table 4).

**Table 4:** Added predictive value of TMAO > 2.26 µmol/L for CV death in PAD patients.

	*TMAO, cutoff (>2.26* *µmol/L)*		
	Value	95% CI^a^	p
Model 2			
Harrell’s C			
Basal model	0.787	0.713 to 0.862	
Basal model + biomarker	0.805	0.737 to 0.873	0.40
IDI	0.034	−0.002 to 0.104	0.19
NRI	0.700	0.169 to 0.949	<0.001
Model 3			
Harrell’s C			
Basal model	0.738	0.652 to 0.823	
Basal model + biomarker	0.760	0.681 to 0.840	0.31
IDI	0.018	−0.006 to 0.074	0.38
NRI	0.667	0.094 to 0.967	0.003
Model 4			
Harrell’s C			
Basal model	0.629	0.492 to 0.767	
Basal model + biomarker	0.683	0.564 to 0.802	0.16
IDI	0.035	0.001 to 0.102	0.17
NRI	0.712	0.362 to 0.953	<0.001

Model 2: sex, age and hs-CRP (log-transformed). Model 3: diabetes mellitus, hypertension, and eGFR (<60 mL/min/1.73 m^2^). Model 4: smoking, dyslipidemia, HDL-C. IDI, integrated discrimination improvement; NRI, net reclassification improvement. ^a^The variance was calculated using bootstrapping (with 1000 resamples) for the IDI and NRI estimates and the jackknife approach for the Harrell’s C estimates.

## Discussion

We determined plasma levels of TMAO to assess their association with PAD severity and their possible use as prognostic markers in symptomatic PAD. TMAO was independently associated to PAD severity and CV-mortality, but not to all-cause death and MACE.

The analysis of circulating gut-microbiome derived products has identified TMAO, a secondary product after TMA is metabolized by the liver enzyme FMO3, as a possible prognostic marker in cardiometabolic diseases^5–7^. In most cases increased levels of TMAO or its initial precursors, betaine, choline or L-carnitine, have been associated to atherosclerotic burden^5,9,10,17^ and worse outcome in arterial pathologies in large cohorts^6,7,11–13^. In addition, the causal role of TMAO in atherosclerosis development has been further studied in murine models, showing accelerated plaque development with dietary supplementation of TMAO or its precursors (e.g.: choline, L-carnitine)^5,11,15,18,19^. Nonetheless, some authors found no predictive value of high TMAO levels for CV events or mortality in smaller cohorts of patients with suspected coronary artery disease^20^ or receiving dialysis^21^, and the contribution of TMAO to early atherosclerosis development in healthy-middle-aged adults is unclear^22^. Symptomatic PAD patients present a complex pathophysiology, frequently associated with comorbidities such as diabetes, hypertension, or CKD, that are suspected to greatly interfere with the metabolism of microbial derived products^8^. Indeed, TMAO homeostasis considerably depends on liver FMO3 activity^16^ and renal clearance^8,23^. We found increased levels of TMAO in CLI patients compared to IC, and also determined hepatic enzymes for liver function assessment, finding no differences in transaminases between IC and CLI. Despite these results, we cannot exclude increased FMO3 activity in our cohort as no direct measurement of the enzymatic activity was performed. Moreover, other factors such as sex hormones, bile acids or insulin have been also described to regulate FMO3 activity and increase TMA oxidation^24,25^.

TMAO accumulation might be also related to worse kidney clearance^23^, since it has been described that patients with decreased kidney function present elevated levels of TMAO in circulation compared to those without CKD^21,26–28^. A trend for reduced eGFR according to TMAO quartiles was reported in the KarMeN study^29^, while no association of TMAO with creatinine was observed in the EPIC-Heidelberg study^30^, suggesting an irrelevant role of the kidney function for TMAO homeostasis in healthy subjects. In contrast, we found a negative correlation between TMAO and eGFR in a small cohort of people with no manifest cardiovascular disease, but with more than two cardiovascular risk factors, and older than those in the studies by Krüger *et al*. and Kühn *et al*.^29,30^. These data suggest that individuals with mildly impaired kidney function might be more susceptible to the detrimental effects of TMAO accumulation. In line with these observations and taking in consideration the high percentage of CKD patients in our PAD population (39%), we found a lineal-inverse association between TMAO and eGFR, and tested the possible interaction between the two according to disease severity. No interaction between TMAO and eGFR was found, however, considering the important role of the kidney on TMAO homeostasis^8^, eGFR was included as covariate for further regression analysis. To assess the accuracy of TMAO levels for PAD patient stratification we established the association between TMAO and PAD severity after adjustment for traditional risk factors and described a cutoff value with diagnostic purposes.

Increased levels of plasma TMAO have been shown to predict future major adverse cardiac events including myocardial infarction, stroke, and death in different CV pathologies^6,7,11,12,31^ and a role for gut derived metabolites in thrombosis has been described *in vivo*, showing that intestinal microbes can directly modulate platelet hyperresponsiveness and clot formation rate via TMAO generation^12,16^. Even if most studies point towards TMAO as marker of worse outcome in CV pathologies, controversy remains when specific patient cohorts are evaluated. For example, no correlation between TMAO and increased risk for CV disease or MACE was observed in a group of end-stage renal disease patients^21^ and in subject with suspected CAD undergoing coronary angiography^20^. Mueller *et al*. speculated that those results could be confounded at least in part by impaired kidney function or poor metabolic control^20^ and encouraged the consideration of these parameters when interpreting the results. In addition, TMAO has been studied mainly in coronary pathologies, while it is prognostic value in other arterial localization, such as PAD, has been little investigated. We report an association between TMAO and CV-mortality, but not with all-cause death or MACE. Our results differ in part from previous data, describing an association of TMAO levels and global death in PAD^14^. However, differences between the studied populations regarding PAD definition and severity status should be considered when interpreting the data. Senthong *et al*. included by the term PAD the majority of non-coronary arterial territories^14^, while our cohort is restricted to symptomatic lower limb artery disease (mean ABI 0.55 ± 0.19). TMAO appears to be better for CV-mortality prognosis than for all-cause death, which might be important for patient evaluation.

### Limitations

The current study including 262 patients could be considered as small, however the high percentage of deaths (39% all-cause and 15% CV origin) provides the statistical power required to support our conclusions. Events were recorded during a mean follow up of 4 years, reasonable to estimate early to medium-term mortality. Longer term studies should be designed to confirm the involvement of TMAO in PAD mortality. No causal relationship between high TMAO levels and CV-mortality can be inferred from our prospective study. Death cause in some patients was unknown and we might have lost some cases related to CV events. Finally, the influence of two important variables for TMAO production, the use of antibiotics and modifications in dietary habits were not recorded and could not be included as confounding variables in our population. FMO3 activity, which has been implicated in thrombosis risk^16^ and converts TMA in TMAO^24^, was not measured.

### Conclusions

We show increased TMAO levels according to PAD severity and an independent association between TMAO and elevated risk for CV-mortality. The design of novel therapeutic strategies towards gut-derived metabolite control in vascular patients will need to consider not only intestinal bacterial function, but also the activity of key hepatic enzymes for TMA oxidation (FMO3), and renal function.

## Methods

The clinical and demographic characteristics of the PAD cohort were previously described by Martinez-Aguilar *et al*.^32,33^. As the cohort has included new cases we include the complete description in supplemental material and methods. Control subjects were previously described by Marcos-Jubilar M *et al*.^34^.

### Baseline characteristics of PAD and control patients

PAD Patients [n = 262, mean age 70 years (SD: 11), 87% men] were prospectively enrolled at the outpatient service of the Department of Vascular Surgery of Complejo Hospitalario de Navarra between 2010 and 2017 (supplemental information). Blood samples were collected at the time of clinical evaluation and tested for biochemical parameters. Ankle brachial index (ABI) was measured at rest, in both lower limbs.

Fontaine classification was used for severity assessment as follows: intermittent claudication (IC, Fontaine class II, n = 147) diagnosed by hemodynamic study (Doppler ultrasound), and critical limb ischemia (CLI, n = 115) with lower limb rest pain and/or trophic lesions (Fontaine class III-IV) confirmed by imaging studies (arteriography, magnetic resonance angiography, or ultrasonography). Exclusion criteria were established as follows: patients with Fontaine class IV and infected-lesions, individuals with evidence of neoplastic disease, generalized or localized inflammatory disease (moderate or severe), severe chronic kidney disease, on haemodialysis, or receiving antinflammatory drugs.

Control subjects (n = 45) were enrolled at the outpatient service of the Department of Internal Medicine, Clínica Universidad de Navarra (April 2016-December 2017). Blood samples were collected at the time of clinical evaluation. Patients were included if older than 45 years, with ≥2 cardiovascular risk factors and no manifested cardiovascular disease at recruitment. Exclusion criteria included active neoplastic disease, acute or chronic inflammatory disease of any aetiology, and intake of nonsteroidal anti-inflammatory or steroid drugs 2 weeks before blood withdrawal. Samples and data from control patients were provided by the Biobank of the University of Navarra and were processed following standard operating procedures approved by the Ethical and Scientific Committees.

The study was approved by the Institutional Review Boards of Complejo Hospitalario de Navarra and Clínica Universidad de Navarra, according to the standards of the Declaration of Helsinki on medical research, and written informed consent was obtained from all patients who were enrolled in this study.

PAD patients were followed up for a mean period of 4 years (min 1 max 102 months) at the outpatient service of the Department of Vascular Surgery every 3, 6 or 12 months, depending on the severity of PAD. Death, either all-cause or cardiovascular, and MACE including amputation, stroke, myocardial infarction and all-cause death were recorded.

### TMAO determination

Based on a previously reported approach by Awwad H.M., *et al*.^35^, a precise and reliable UHPLC-MS/MS method has been implemented in our laboratory for the quantification of TMAO in human plasma. Frozen citrate plasma samples, in which the corresponding stabilized TMA salt was formed, were utilized to perform these analyses. Concentrations of the analyte in plasma samples was determined from calibration curves, which indicated a good linearity (r^2^ ≥ 0.999) within the studied concentration range (100 nM–10 μM), using peak area ratio of the analyte to its isotope. The implemented UHPLC-MS/MS method has good analytical accuracy, precision and recovery; in fact, the recoveries ranged between 97% and 104% and, accuracy and precision of the assays were between 3.7% and 7.3% (reported in the Supplementary Table S1). Details about standards and chemicals, calibration curve and sample preparation as well as UHPLC and MS system conditions are explicitly described in the Supplementary Information together with their corresponding Figures (Supplementary Figs S1 and S2).

### Statistical analysis

Normality was demonstrated by the Kolmogorov-Smirnov test. Non-normally distributed variables were log-transformed. Differences between two groups of subjects were tested by Student’s t test (normal unpaired data) or Mann-Whitney U test (nonparametric test). χ2 or Fisher’s exact test was used for categorical variables. Association studies were performed by Pearson correlation test for continuous variables. Receiver Operating Characteristic (ROC) curves were plotted to assess disease severity (IC vs. CLI), and the cut-off value for TMAO established with the Youden Index. Multivariable logistic regression models were adjusted for relevant covariates: age, sex, cigarette smoking, diabetes mellitus, hypertension, dyslipidemia, HDL-C, eGFR (<60 mL/min/1.73 m^2^) and hs-CRP^14,32^. Multicollinearity was evaluated by the variance inflation factor and models calibrated by the Homer-Lemeshow goodness-of-fit test. Patients without outcome were censored at the date of their last follow-up. Hazard ratios (HR) and their 95% CI for death (all-cause) and MACE were estimated using Cox regression models after adjusting for relevant covariates. Fine-Gray competing risk models were used to obtain sub-hazard ratios for CV death, considering non-CV death as a competing event. Due to the low number of CV death events, the following basal models were considered for adjustment for relevant covariables: model 1, unadjusted; model 2, sex and hsCRP (log); model 3, diabetes mellitus, hypertension and eGFR (<60 mL/min/1.73 m^2^); model 4, smoking, dyslipidemia and HDL-C. The additional value of TMAO for risk prediction of CV death was assessed with Harrell’s C statistics and the continuous net reclassification index (NRI) index. Values are expressed as mean ± SD or median (interquartile range), and categorical variables as numbers and percentages. Analyses were performed with STATA version 12 (Stata Corp., College Station, TX, USA) and SPSS version 15. All p-values are two-tailed, and statistical significance was set at p < 0.05.

## Supplementary information

Supplemental material SREP-19-23703A

## Data Availability

The datasets generated during and/or analysed during the current study are available from the corresponding author on reasonable request.
