# Supplementary material for: Trimethylamine-N-Oxide (TMAO) Predicts Cardiovascular Mortality in Peripheral Artery Disease
Source: Sci Rep. 2019 Oct 30;9:15580. doi: 10.1038/s41598-019-52082-z (PMC6821861; doi:10.1038/s41598-019-52082-z)
Supplement: Supplementary file 1 — Supplemental material SREP-19-23703A [file 41598_2019_52082_MOESM1_ESM.pdf]

SUPPLEMENTAL INFORMATION

TRIMETHYLAMINE-N-OXIDE (TMAO) PREDICTS CARDIOVASCULAR MORTALITY IN PERIPHERAL ARTERY DISEASE

Carmen Roncal PhD, Esther Martínez-Aguilar MD, PhD, Josune Orbe PhD, Susana Ravassa PhD, Alejandro Fernandez-Montero PhD, Goren Saenz-Pipaon Msc, Ana Ugarte PhD, Ander Estella-Hermoso de Mendoza PhD, Jose A. Rodriguez PhD, Sebastián Fernández-Alonso MD, Leopoldo Fernández-Alonso MD, PhD, Julen Oyarzabal PhD, Jose A. Paramo MD, PhD

SUPPLEMENTARY METHODS

The clinical and demographic characteristics of the PAD cohort were previously described by Martinez-Aguilar et al [1,2]. As the cohort has included new cases we include the complete description of the methods. Control subjects were previously described by Marcos-Jubilar M et al [3].

Baseline characteristics of patients

Patients [n=262, mean age 70 years (SD: 11), 87% men] were prospectively enrolled and blood samples collected at the time of clinical evaluation at the outpatient service of the Department of Vascular Surgery of the Complejo Hospitalario de Navarra between 2010 and 2017.

Patients were classified according to the severity of the disease in intermittent claudication (IC, Fontaine class II, n=147) diagnosed by hemodynamic study (Doppler ultrasound), and critical limb ischemia (CLI, n=115) with lower limb rest pain and/or trophic lesions (Fontaine class III-IV) confirmed by imaging studies (arteriography, magnetic resonance angiography, or ultrasonography). Among those patients belonging to Fontaine class IV the ones with infected-lesions were excluded from the study, as well as individuals who had evidence of neoplastic disease, generalized or localized inflammatory disease (moderate or severe), severe chronic kidney disease, on haemodialysis, or receiving antinflammatory drugs. Ankle brachial index (ABI) was measured at rest, as per standard technique in the dorsalis pedis and posterior tibial arteries of both lower limbs [4].

A thorough medical listing was recorded in all patients including history of previous myocardial infarction, non-ischemic cardiomyopathy, cerebrovascular disease,

diabetes mellitus, arterial hypertension, smoking status, and medication. Patients were considered smokers (at least 1 cigarette daily on average over the past year), never smokers, and former smokers (no cigarette consumption in the past year). Diabetes was defined by history of diabetes mellitus or the use of antidiabetic drugs. Hypertension was defined by any history of hypertension or the use of antihypertensive drugs. Dyslipidemia was defined as increased plasma concentrations of cholesterol and/or triglycerides or the use of lipid lowering drugs.

Control subjects (n=45) were enrolled at the outpatient service of the Department of Internal Medicine, Clínica Universidad de Navarra (April 2016-December 2017). Blood samples were collected at the time of clinical evaluation. Patients were included if older than 45 years, with ≥2 cardiovascular risk factors and no manifested cardiovascular disease at recruitment. Exclusion criteria included active neoplastic disease, acute or chronic inflammatory disease of any aetiology, and intake of nonsteroidal anti-inflammatory or steroid drugs 2 weeks before blood withdrawal. Samples and data from control patients were provided by the Biobank of the University of Navarra and were processed following standard operating procedures approved by the Ethical and Scientific Committees.

The study was approved by the Institutional Review Boards of the Complejo Hospitalario de Navarra and Clínica Universidad de Navarra, according to the standards of the Declaration of Helsinki on medical research, and written informed consent was obtained from all patients who were enrolled in this study. No patient was included post-operatively.

Follow-up

PAD patients were followed up for a mean period of 4 years (min 1 max 102 months) at the outpatient service of the Department of Vascular Surgery every 3, 6 or 12 months, depending on the severity of PAD. At recruitment patients underwent physical examination and ABI assessment and were tested for biochemical parameters. No patient was lost to the follow-up. For outcome evaluation of PAD patients, death, either all-cause or cardiovascular, and Major adverse cardiovascular events (MACE) including amputation, stroke, myocardial infarction and all-cause death were recorded at the exact date of the event.

Laboratory analysis

Serum total cholesterol, HDL cholesterol, triglycerides and glucose were measured in fasting blood samples by standard laboratory techniques. LDL cholesterol was estimated using the Friedewald equation. Plasma fibrinogen activity was measured by clotting assay (Clauss) and high-sensitivity (hs)-CRP by immunoassay (Immulite; Diagnostic Product Corporation).

TMAO determination

Standards and chemicals

TMAO (Sigma–Aldrich). D9-TMA DCl and d9-TMAO (Cambridge Isotope Laboratories) were used as internal standards. Ammonium formate (Sigma–Aldrich) and formic acid for mass spectrometry (Fluka). HPLC-MS grade acetonitrile (Merck) and methanol (Scharlau). Ultrapure water (18.2MΩ, Milli-Q water purification system, Millipore).

Calibration curve and sample preparation

Frozen citrate plasma samples, in which the corresponding stabilized TMA salt had been formed, were thawed before analysis and vortex mixed. Quantification was achieved by external calibration using matrix-matched standards. Calibration standards were prepared by adding the appropriate volume of diluted solutions of the analytes (mixture of methanol and water, 50:50, v:v) to aliquots of 50µL of blank plasma. The calibration standard and sample preparation: each 50µL of plasma or calibration standards were mixed with 10µL of internal standard working solution containing a mixture of d9-TMAO in methanol. To precipitate the proteins, 200µL of 3% formic acid spiked-methanol were added to the samples. After brief vortex-mixing, samples were subjected to centrifugation at 20,000×g for 20min at 10°C, and supernatants were transferred into 2mL vials for analysis.

A calibration curve (seven calibrators and a blank) was prepared for dTMAO, covering a range between 100nM and 10µM, to examine the linearity. The linearity of the calibration curves was evaluated using the coefficient of determination (r^2) and the percentage of deviation (%dev) for each sample. The obtained r^2 were ≥0.999, ≤10% deviation in all cases, which indicates a good linearity within the studied concentration range (Supplementary Figure S1).

UHPLC and MS system conditions

Quantification of TMAO and corresponding isotope was performed using an ACQUITY UPLC system coupled with a Xevo TQ Detector mass spectrometer (UPLC-MS/MS) (Waters, Milford, MA, USA). Chromatographic separation was

performed on an XBridge BEH Amide XP column, 130Å, 2.5µm, 2.1mmx50mm from Waters (Milford, MA, USA). The column was heated to 30°C and the flow rate maintained at 0.4 mL/min. Samples were separated using a gradient mobile phase that consisted of a mixture of 10mM ammonium formate (pH 3.5) as solvent A and acetonitrile as solvent B. Gradient conditions were: 0.0–0.5 min, 10–30% A; 0.5–1 min, 30% A; 1–1.4min, 30–60% A; 1.4–1.80min, 60% A; 1.80–1.81min, 60–90% A; 1.81–3min, 90% A; 3–3.1min, 90–10% A; and 3.1–5.0min, 10% A. Total runtime was 5min.

The detection was performed using positive electrospray ionization mode in multiple reaction monitoring (MRM) mode. Electrospray ionization voltage was 2.8kV. Nitrogen was used as a desolvation gas at 300L/min and as an auxiliary gas in the cone at 20L/min. The temperature of the source was 150°C and the desolvation temperature was 300°C. Argon was used as a collision gas at a rate of 1mL/min. Cone voltage and collision energy for each were 30V for TMAO; and 20V and 24V for d9-TMAO. The analyte and its corresponding isotope were monitored using characteristic ion transitions: 76>58 for TMAO, 85>66 for d9-TMAO (examples of MRM chromatograms reported as Supplementary Figure S2). Concentration of the analyte in samples was determined from calibration curves using peak area ratio of the analyte to its isotope. Then, the recovery of the assay was investigated by spiking the plasma with three different concentrations (500nM, 2.5µM and 5µM) of TMAO [5].The recoveries ranged between 97% and 104% for TMAO, and accuracy and precision of the assay was between 3.7% and 7.3% (reported in the Supplementary Table S1).

Statistical analysis

Normality was demonstrated by the Kolmogorov-Smirnov test. Non-normally distributed variables were logarithmically transformed. Differences between two groups of subjects were tested by Student's t test (normal unpaired data); otherwise, the nonparametric test (Mann-Whitney U test) was used. $\chi 2$ or Fisher's exact test was used for categorical variables.

Association studies were performed by Pearson correlation test for continuous variables. Receiver Operating Characteristic (ROC) curves were plotted to assess disease severity (IC vs. CLI) and the cut-off value for TMAO established with the Youden Index. Multivariable logistic regression models for disease severity and TMAO were adjusted for relevant covariates: age, sex, cigarette smoking, diabetes mellitus, hypertension, dyslipidemia, HDL-C, eGFR (<60 mL/min/1.73m^2) and hs-CRP [1,6]. Multicollinearity was evaluated by the variance inflation factor and models calibrated by the Homer-Lemeshow goodness-of-fit test.

Patients without outcome were censored at the date of their last follow-up. Hazard ratios (HR) and their 95% CI for death (all-cause) and MACE were estimated using COX regression models after adjusting for relevant covariates. Fine-Gray competing risk models were used to obtain sub-hazard ratios for CV death, considering non-CV death as a competing event. Due to the low number of CV death events, the following basal models were considered for adjustment for relevant covariables: model 1, unadjusted; model 2, sex and hsCRP (log); model 3, diabetes mellitus, hypertension and eGFR (<60 mL/min/1.73m2); model 4, smoking, dyslipidemia and HDL-C. The proportional sub-hazard and hazard

assumptions were verified using Schoenfeld's residuals for each model. If violated, standard Cox or competitive risk regression analyses were extended including time varying covariates for each variable that did not satisfy this assumption.

The additional value of TMAO for risk prediction of CV death was assessed with Harrell's C statistics and the continuous net reclassification index (NRI) index. The variance for Harrell's C estimates was calculated using the jackknife approach with the Stata package "somersd". The variances for the NRI estimates were calculated using bootstrapping (1000 resamples) with the Stata command "incrisk".

Values are expressed as mean±SD or median (interquartile range), and categorical variables as numbers and percentages. Analyses were performed with STATA version 12 (Stata Corp., College Station, TX, USA) and SPSS version 15. All p-values are two-tailed, and statistical significance was set at P<0.05.

SUPPLEMENTARY REFERENCES

1. Martinez-Aguilar, E. *et al.* Matrix metalloproteinase 10 is associated with disease severity and mortality in patients with peripheral arterial disease. *J Vasc Surg* **61**, 428–435 (2015).

2. Martinez-Aguilar, E. *et al.* Reduced high-density lipoprotein cholesterol: A valuable, independent prognostic marker in peripheral arterial disease. *J. Vasc. Surg.* **66**, 1527–1533 (2017).

3. Marcos Jubilar, M. *et al.* Analysis of monocitary subpopulations in relation to cardiovascular risk factors. *Clin. Investig. Arterioscler.* **31**, 152–159 (2019).

4. Hendriks, E. J. E. *et al.* Association of High Ankle Brachial Index With Incident

Cardiovascular Disease and Mortality in a High-Risk PopulationSignificance. *Arterioscler. Thromb. Vasc. Biol.* **36**, 412–417 (2016).

5. Awwad, H. M., Geisel, J. & Obeid, R. Determination of trimethylamine, trimethylamine N-oxide, and taurine in human plasma and urine by UHPLC–MS/MS technique. *J. Chromatogr. B* **1038**, 12–18 (2016).

6. Senthong, V. *et al.* Trimethylamine N-Oxide and Mortality Risk in Patients With Peripheral Artery Disease. *J. Am. Heart Assoc.* **5**, e004237 (2016).

SUPPLEMENTARY FIGURES

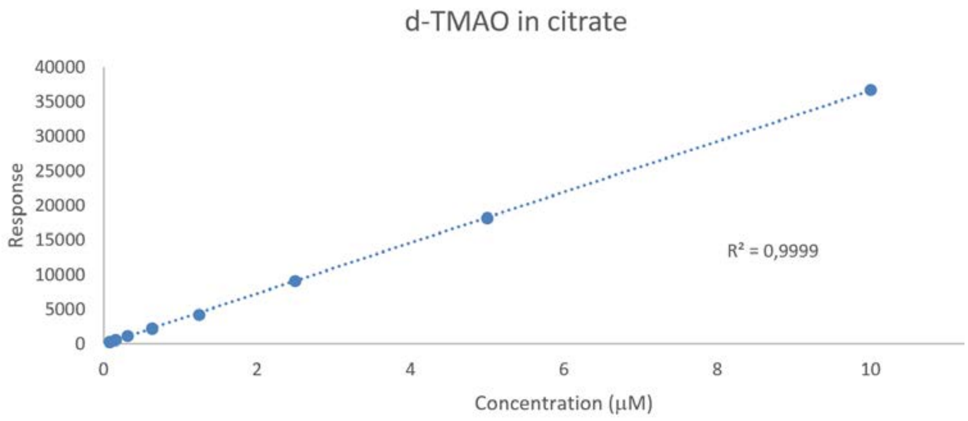

Supplementary Figure 1. TMAO calibration curves. dTMAO: d9trimethylamine N-oxide.

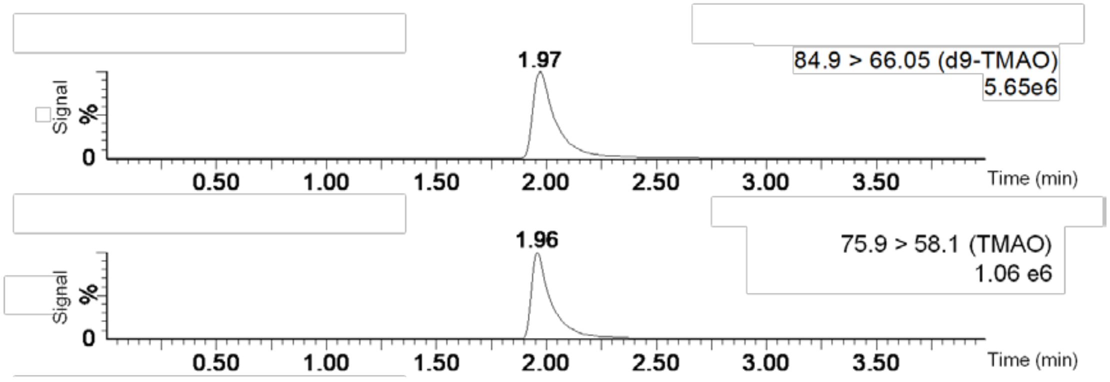

Supplementary Figure 2. Example of MRM chromatograms: TMAO and their internal standards obtained in plasma samples by UHPL-MS/MS. Transitions of m/z and peak intensities are shown in the upper right corner.

SUPPLEMENTARY TABLES

Supplementary Table 1. Analytical accuracy and precision of the assay for the quantitation of analytes in plasma.

	Spiked with 0.5 µmol/L				Spiked with 2.5 µmol/L				Spiked with 5 µmol/L			
	Measured mean (SD) µmol/L	Accuracy (%)	CV (%)	Recovery (%)	Measured mean (SD) µmol/L	Accuracy (%)	CV (%)	Recovery (%)	Measured mean (SD) µmol/L	Accuracy (%)	CV (%)	Recovery (%)
dTMAO Plasma	0.49 (0.03)	2	4.8	98	2.58 (0.10)	-3.2	3.8	103.2	5.10 (0.29)	-2	4.4	102

Table S1. Accuracy and precision of the assay was determined by replicate measurements (n = 6) of quality control samples at three concentration levels of analyses. The accuracy was expressed as (measured concentration − theoretical concentration)/(theoretical concentration) × 100 and the precision by the CV (%) of the measured concentration values obtained after analysis of the quality control samples with different nominal concentration values. Recovery was expressed as (measured concentration)/(theoretical concentration) × 100.SD: Standard deviation:, dTMAO: d9 trimethylamine N-oxide, CV: Coefficient of variation and n=6 for each spiked concentration.

Supplementary Table 2. Demographic and clinical parameters of control subjects.

	Control (n=45)
Sex (male, %)	67
Age (years)	68(7)
Smokers (%)	
Never	40
Current	16
Former	44
Diabetes mellitus (%)	29
Hypertension (%)	58
Dyslipidemia (%)	78
BMI (kg/m^2)	29(4)
Laboratory data	
Total col (mg/mL)	185(38)
LDL-C (mg/dL)	103(39)
HDL-C (mg/dL)	60(16)
Triglycerides (mg/dL)	108(46)
hs-CRP[a] (mg/mL)	1.2(2)
AST (U/L)	22(5)
ALT (U/L)	25(10)
GGT (U/L)	40(41)
eGFR ($mL/min/1.73m^2$)	82(10)

Mean (SD) is shown. [a]Log-transformed variables are presented as median (Interquartile range). BMI: body mass index, LDL: low-density lipoprotein, HDL: high-density lipoprotein. ALT: alanine aminotransferase; AST: aspartate aminotransferase; GGT: gamma glutamyltransferase; eGFR: estimated Glomerular filtration rate.

Supplementary Table 3. Association of TMAO (µmol/L) with all-cause death and MACE.

	TMAO, µmol/L[a]		
	HR	95% CI	P value
All-cause death			
Model 1	1.30	1.13-1.46	<0.001
Model 2	1.09	0.94-1.26	0.236
MACE			
Model 1	1.28	1.15-1.44	<0.001
Model 2	1.08	0.95-1.23	0.199

Hazard ratios (HR) are effects sizes for a doubling of TMAO in plasma. Model 1: unadjusted. Model 2: sex, age, smoking, diabetes mellitus, hypertension, dyslipidemia, HDL-C, eGFR (<60 mL/min/1.73m^2), and hs-CRP (log). [a]Log-transformed variable.
